# Ankyrin-B Syndrome: Enhanced Cardiac Function Balanced by Risk of Cardiac Death and Premature Senescence

**DOI:** 10.1371/journal.pone.0001051

**Published:** 2007-10-17

**Authors:** Peter J. Mohler, Jane A. Healy, Hui Xue, Annibale A. Puca, Crystal F. Kline, R. Rand Allingham, Evangelia G. Kranias, Howard A. Rockman, Vann Bennett

**Affiliations:** 1 Department of Internal Medicine, University of Iowa Carver College of Medicine, Iowa City, Iowa, United States of America; 2 Department of Molecular Physiology and Biophysics, University of Iowa Carver College of Medicine, Iowa City, Iowa, United States of America; 2 Howard Hughes Medical Institute, Duke University Medical Center, Durham, North Carolina, United States of America; 3 Department of Cell Biology, Duke University Medical Center, Durham, North Carolina, United States of America; 4 Department of Biochemistry, Duke University Medical Center, Durham, North Carolina, United States of America; 5 Department of Medicine, Duke University Medical Center, Durham, North Carolina, United States of America; 6 Department of Ophthalmology, Duke University Medical Center, Durham, North Carolina, United States of America; 7 Istituti di Ricovero e Cura a Carattere Scientifico (IRCCS) Policlinico MultiMedica, Milan, Italy; 8 Department of Pharmacology and Cell Biophysics, University of Cincinnati College of Medicine, Cincinnati, Ohio, United States of America; The Research Institute for Children, United States of America

## Abstract

Here we report the unexpected finding that specific human *ANK2* variants represent a new example of balanced human variants. The prevalence of certain *ANK2* (encodes ankyrin-B) variants range from 2 percent of European individuals to 8 percent in individuals from West Africa. Ankyrin-B variants associated with severe human arrhythmia phenotypes (eg E1425G, V1516D, R1788W) were rare in the general population. Variants associated with less severe clinical and *in vitro* phenotypes were unexpectedly common. Studies with the ankyrin-B^+/−^ mouse reveal both benefits of enhanced cardiac contractility, as well as costs in earlier senescence and reduced lifespan. Together these findings suggest a constellation of traits that we term “ankyrin-B syndrome”, which may contribute to both aging-related disorders and enhanced cardiac function.

## Introduction

Ankyrins -R, -B, and -G are a versatile family of membrane-associated adaptors that were first discovered based on the role of ankyrin-R in mediating membrane attachment of spectrin in human erythrocytes [1]–[3]. Consistent with this function, mutations in ankyrin-R are the major cause of hereditary spherocytosis, a disorder associated with spectrin deficiency and increased membrane fragility in humans [4]–[6]. Ankyrins-G and -B are expressed in non-erythroid tissues, where they function in the maintenance and establishment of specialized membrane domains by interacting with a diverse set of membrane proteins. Ankyrin-G is required for accumulation of voltage-gated sodium channels in excitable membranes at axon initial segments of neurons [7], [8] and at transverse-tubules in cardiomyocytes [9]. A cardiac voltage-gated sodium channel *SCN5A* human variant that abolishes binding of Na_v_1.5 with to ankyrin-G results in Brugada syndrome, which is a dominantly-inherited fatal cardiac arrhythmia [9]. Ankyrin-G also is required for biogenesis of the lateral membrane of epithelial cells and pre-implantation embryos [10], [11]. Loss of ankyrin-G function thus would likely have major consequences beginning in early development. In contrast to ankyrin-G, ankyrin-B has more specialized roles, and is required for postnatal life in mice but not for prenatal survival [12].

Loss-of-function variants in *ANK2* (encodes ankyrin-B) cause a dominantly-inherited cardiac arrhythmia with increased risk for sudden cardiac death, initially termed type 4 long QT syndrome, and more recently renamed sick sinus syndrome with bradycardia or the “ankyrin-B syndrome” ([13]–[15]; OMIM). Mice heterozygous for a null mutation in ankyrin-B have a similar arrhythmia to humans and exhibit sudden death following administration of catecholamines and/or exercise [14]. Studies with ankyrin-B^+/−^ cardiomyocytes revealed that the basis for sudden death associated with the arrhythmia is elevation of calcium transients in the context of sympathetic stimulation [14]. The cellular basis for altered calcium dynamics in ankyrin-B^+/−^ cardiomyocytes has been proposed to result from a deficiency of a complex of ankyrin-B with the Na/K ATPase, Na/Ca exchanger, and IP3 receptor localized in a specialized microdomain in cardiomyocyte transverse-tubules [16]. Loss of additional signaling molecules from ankyrin-B^+/−^ myocytes may also influence the cellular phenotype in ankyrin-B deficiency [17].

Abnormal calcium dynamics in ankyrin-B^+/−^ neonatal cardiomyocytes can be rescued by expression of wild-type human ankyrin-B but not by ankyrin-B with variants associated with cardiac arrhythmia [13], [14], [18]. These ankyrin-B variants demonstrate clear functional differences *in vitro*. However, similar to other inherited arrhythmia syndromes [19], [20], the penetrance of the sudden cardiac death phenotype in families with ankyrin-B variants can be relatively low. For example, in the first family studied with the E1425G variant, while numerous individuals displayed sinus node dysfunction, atrial fibrillation, and ventricular arrhythmia, only 2 of the 21 heterozygotes experienced fatal outcomes [14]. Moreover, specific ankyrin-B variants associated with minor cellular phenotypes may be relatively common in specific human populations [21].

Here, we present evidence that specific ankyrin-B variants represent a new example of balanced variants in humans. The prevalence of ankyrin-B variants varied from 2 percent of European to 8 percent in individuals from West Africa. Moreover, African and European populations also exhibit distinct patterns of ankyrin-B variants. Studies with the ankyrin-B^+/−^ mouse reveal both benefits of enhanced cardiac contractility, as well as costs in earlier senescence and reduced lifespan. Together these findings suggest a constellation of traits that we term “ankyrin-B syndrome”, which may contribute to both aging-related disorders and enhanced cardiac function.

## Materials and Methods

### Protein Alignment

Protein alignments were performed in CLUSTALW using the following protein sequences from NCBI: *Homo sapiens* gi|119626696|gb|EAX06291.1; *Macaca mulatta* gi|109075425|ref|XP_001095471.1; *Canis familiaris* gi|74002173|ref|XP_545031.2; *Mus musculus* gi|37590265|gb|AAH59251.1|; *Rattus norvegicus* gi|109467596|ref|XP_001076082.1|; *Pan troglodytes* gi|114595754|ref|XP_517403.2; *Gallus gallus* gi|118090374|ref|XP_420641.2.

### Genetic Studies

*ANK2* variants reported previously to have functional consequences in cardiomyocytes were used for genotyping genomic DNA samples from West Africans residing in the country of Ghana (collected by Rand Allingham and co-workers, Duke University Dept. of Ophthalmology) and Europeans (collected by the Associazone Longevita, Italy). SNP genotyping was determined using the ABI 7900HT Taqman SNP genotyping system (Applied Biosystems, Foster City, California, United States), which uses a PCR-based allelic discrimination assay in a 384-well–plate format with a dual laser scanner. Allelic discrimination assays were purchased from Applied Biosystems, or, if the assays were not available, primer and probe sets were designed and purchased through Integrated DNA Technologies (Coralville, IA,). Successful genotyping was obtained for greater than 95% of the DNA samples used in the study.

### Animal care

Ankyrin-B^+/−^ mice [12] were backcrossed >10 generations (>99.5% pure) into a C57/Bl6 background before experiments. Both ankyrin-B^+/−^ and wild-type littermates mice were housed in the same facility (temperature and humidity), consumed the same diet (Lab Diet, 23% protein, 4.5% fat, 6.0% fiber, 8.0% ash, 2.5% minerals (0.95% Ca2+, 0.67% phosphorus, 0.40% non-phytate phosphorus), 56% complex carbohydrate from overhead wire feeders) and water ad libitum, and were kept on identical 12 hour light/dark cycle. Food intake was monitored over 24 hour periods and averaged over 2–3 days in mice individually housed.

### ^3^H ouabain-binding

Equilibrium [^3^H] ouabain binding was performed on isolated live adult cardiomyocytes (>70% rod-shaped) for 60 min at 35°C in modified Tyrode's buffer (0.5 mM KCl). Non-specific binding was determined as binding in the presence of 10 mM non-radioactive ouabain. Membranes were separated from buffer and sequentially washed by vacuum filtration in cold buffer followed by liquid scintillation counting.

### Mouse echocardiograms

Two-dimensional guided M-mode echocardiography was performed using an HDI 5000 echocardiograph (ATL, Bothell, WA) as previously described [22]–[25]. Mice were studied in a conscious state using gentle manual restraint after a period of acclimation [23]. A soft plastic collar was fashioned to prevent the animals from biting the probe. Mice were studied in a conscious state. For pressure overload induced cardiac failure, mice were anesthetized, intubated; the chest was opened in the second intercostal space. A 7-0 suture was placed around the transverse aorta and tied to a 27 gauge needle to create aortic constriction [23], [26]. The needle was then recovered, mice were allowed to recover, and follow-up echocardiograms were performed on wild-type and ankyrin-B^+/−^ mice seven days post-ligation as described [23], [26]. Fractional shortening values were compared between wild-type and ankyrin-B^+/−^ mice over a range of similar pressure gradients. Values represent mean±standard error.

### Mouse studies

Animal care was in accordance with institutional guidelines. Wild-type (n = 6) and ankyrin-B^+/−^ mice (n = 6) were implanted with radiotelemetry transmitters [14]. Following an intraperitoneal injection of a low (100 µg/kg) or high dose (10 mg/kg) of ouabain, or 2 mg/kg epinephrine conscious mice were monitored for heart rate, syncope, or death.

### Preparation of Adult Cardiac Myocytes and Contractility Studies

Mice used in these studies were female adult WT C57BL/6 mice and ankyrin-B^+/−^ littermates (C57 BL/6), or wild-type and PLN^−/−^ littermates (129), 3–6 months of age and weighing 30–40 g. Animals were handled according to approved protocols and animal welfare regulations of the Institutional Review Board (DUMC). Mouse ventricular cardiomyocytes were isolated as described in [27]. Myocytes were isolated and visualized with an inverted microscope (Nikon Eclipse TE 300). Single-cell contraction was measured by video edge detection, and recordings were made under basal conditions and after administration of 100 µM ouabain [27]. Cellular shortening was measured for 15–20 cells of equal length/mouse to determine the mean cellular shortening per mouse under each condition. Values reported represent mean±standard deviation of cellular shortening for 4–5 mice.

### Temperature measurements

Following anesthesia, six month mice were surgically implanted with radiotelemetry temperature probes (Data Sciences International). Five days after surgery, the temperature of non-anesthetized animals was determined.

### Immunofluorescence and Immunoblotting

Freshly extracted pancreas from wild-type C57B/6 and ankyrin-B^+/−^ C57B/6 mice was fixed for 18 hours at 4°C in 4% paraformaldehyde. Tissues were embedded and sectioned at 10 µm thickness. Primary antibody incubation was performed for 18 hours at 4°C and slides were washed thoroughly with a solution of phosphate buffered saline, pH 7.4 (PBS) +0.1% Triton X-100 (v/v). Incubation with secondary antibodies was performed for six hours at 4°C and slides were again washed with PBS +0.1% (v/v) Triton X-100. Pancreas sections were analyzed using the following antibodies: insulin (Santa Cruz, Chemicon) glucagon (Sigma), somatostatin (Chemicon), and ankyrin-B.

### Antibodies

Primary antibodies included rabbit anti-insulin (Santa Cruz Biotechnology); rabbit anti-somatostatin (Chemicon International); mouse anti-glucagon (Sigma); affinity-purified ankyrin-B monoclonal and polyclonal Ig. Secondary antibodies included goat anti-rabbit and goat anti-mouse Alexa Fluor 488 and 568 (Molecular Probes).

### Survival Curve

Ankyrin-B^+/−^ mice (n = 100) and their wild-type littermates (n = 100) (C57B/6) were monitored for three years. Each group of mice were kept in identical environments. Relative survival for each genotype was calculated as a per cent cumulative survival of its group (either wild-type or ankyrin-B^+/−^). Evaluation continued until all mice within each population died (0% survival).

### Immunoblotting

Immunoblots were performed as previously described[12]. Primary antibody incubation was performed for 18 hours at 4°C with gentle rocking. Blots were rinsed using three 15-minute washes in 1× TTBS. Immunoblotting was performed as described [12] using ^125^I-labelled Protein A. Signals were quantitated by phosphorimaging.

### Statistics

Data were analyzed using either paired two-tailed *t* tests or two-way ANOVA, and *P* values less than 0.05 were considered significant (*).

## Results

### Prevalence of ankyrin-B variants in human populations

**Figure 1:**
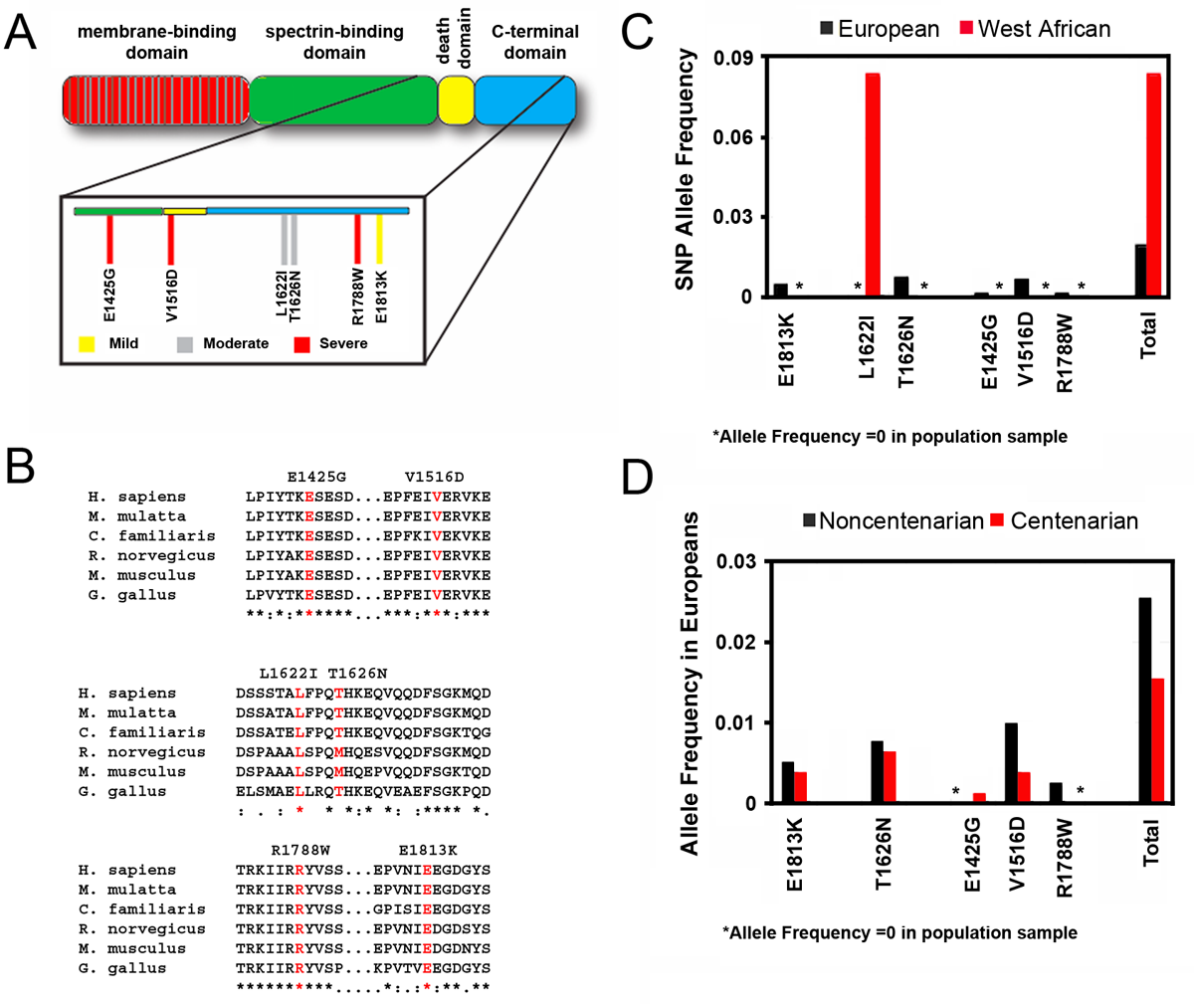
Human ankyrin-B gene variants have unique distributions in distinct ethnic populations and may affect longevity. A. The domain structure of 220 kD ankyrin-B protein, showing the N-terminal membrane binding domain (red), spectrin-binding domain (green), death domain (yellow), and C-terminal domain (blue). Magnified insert shows the location of previously identified variants. Variants had range of functional severity in mouse cardiomyocytes, indicated with yellow (mild), gray (moderate) or red (severe) bars. B. Affected residues are highly conserved across species. Ankyrin-B alignment from human, monkey, rat, mouse, dog, chicken, and zebrafish. Residues affected by the nucleotide variants are shown in red. C. Allele frequency of the *ANK2* variants in 1152 Europeans (black bars) and 384 West Africans (red bars) as determined by PCR based allelic discrimination assay. Right, Allele frequency of pooled functional variants. D. Ankyrin-B variants are less frequent in Europeans that reach the age of 100. Allele frequency of the each variant in European non-centenarians (black bars) and centenarians (red bars). Asterisks indicate that no individuals were identified with the variant.

An initial screen for ankyrin-B variants in arrhythmia patients detected individuals with several mis-sense variants in the ankyrin-B gene [14], [15]. These variants include E1425G, L1622I, T1626N, R1788W, and E1813K [14], [15]. Comparison of ankyrin-B protein primary sequence from different species (Figure 1B) shows that the identified variants are highly conserved in vertebrates. Of the six variants shown, only T1626N does not show 100% sequence identity from humans to chickens. The high degree of conservation across species suggests that these residues are likely to be critical for ankyrin-B stability and/or function. It was therefore unexpected when Sherman and colleagues reported that certain ankyrin-B gene variants were present in healthy Caucasian and African American individuals [21]. Notably, L1622I was found in 4% of African-Americans in this study. One intriguing possibility is that the apparent discrepancy is due to ethnic differences in ankyrin-B SNP allele frequency.

Thus, we first determined the allele frequencies of these genetic variants in defined ethnic populations. We decided to explore the differences between white and black populations. In order to do this, we obtained genomic DNA samples from European and African populations. Since African-Americans represent an admixture of Caucasian, African, and Native American haplotypes that would confound our analysis, we chose to exclude them from this initial study.

We genotyped 384 west African and 1152 European individuals using a PCR-based allelic discrimination assay. Figure 1c shows the allele frequency of each variant in the European (black) and west African (red) populations tested, as well as the allele frequency of the pooled functional variants (Total). L1622I was surprisingly common in Africans, and was present in ∼8.6% of the individuals genotyped. It was the only variant detected in the west African population. In the European population, all SNPs had an allele frequency of less than 0.01, though combined, 2.1% of the European sample had a loss of function ankyrin B variant.

As discussed later, ankyrin-B^+/−^ mice demonstrate signs of accelerated senescence compared to litter matched controls (see below). Using population tools, we next evaluated whether there were negative consequences for longevity in individuals with ankyrin-B variants. If so, we would predict these variants to be depleted or absent in centenarians (humans that reach the age of 100). The European genomic DNA samples used in the population study were a collection of 768 centenarians and 384 non-centenarians obtained from the Associazone Longevita in Italy. Figure 1D shows the allele frequency of the each variant in non-centenarians (black) and centenarians (red). Centenarians had a reduced frequency of variants at 1.6 percent compared to non-centenarians with 2.4 percent. However, the association between ankyrin-B variants and centenarian status was not significant (for combined ankyrin-B loss-of-function variants p = 0.3570 for Fischer's exact test, p = 0.3501 for chi-squared test). However, we believe that the high p value is likely to result from a lack of power at this sample size. Given the low allele frequency of these variants, the p value could be improved by increasing the numbers of individuals tested in each group. At the proportions seen in this study, we would need approximately 6000 patients to get a significant p value. We hope to address this study limitation in the future.

These data indicate that specific ankyrin-B gene variants are both more common than expected and segregated with respect to ethnicity. Negative consequences for those having a function ankyrin-B variant may include both susceptibility to cardiac arrhythmia and reduced longevity. However, the prevalence of these variants and racial differences detected in their frequency suggest the interesting possibility that these ankyrin-B variants may benefit organism survival.

### Increased contractility of ankyrin-B^+/−^ cardiomyocytes

We next explored whether ankyrin-B haploinsufficiency could provide a benefit to the mouse. Ankyrin-B^+/−^ cardiomyocytes exhibit a 16 percent elevation in calcium transients [14], which has been proposed to result from loss of Na/K ATPase localized in a complex with ankyrin-B as well as the Na/Ca exchanger and InsP_3_ receptor [16]. Elevated intracellular calcium transients in ventricular cardiomyocytes would lead to enhanced contractility, which would increase cardiac output. These findings suggested the hypothesis that ankyrin-B deficiency mimics effects of cardiac glycosides, which inhibit the Na/K ATPase, cause elevation in intracellular calcium stores, and increase cardiac contractility as a positive outcome.

**Figure 2:**
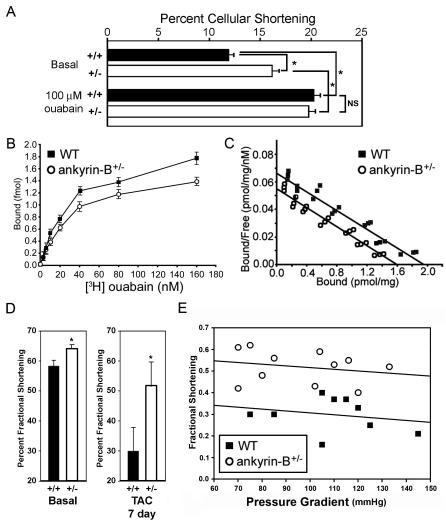
Loss of ankyrin-B-dependent [^3^H]-ouabain-binding sites mimics the cellular action of digitalis. (A) Isolated unloaded ankyrin-B^+/−^ ventricular cardiomyocytes display increased cellular shortening compared to unloaded WT cardiomyocytes, consistent with increased [Ca^2+^]_i_ transients [14]. Asterisk represents p<0.01 between untreated WT and ankyrin-B^+/−^ cardiomyocytes (15–20 cardiomyocytes from 4–5 mice). WT and ankyrin-B^+/−^ cardiomyocytes treated with maximal doses of ouabain (100 µM) display significant elevations in cellular shortening compared with untreated WT and ankyrin-B^+/−^ cardiomyocytes (15–20 cardiomyocytes from 4–5 mice, p<0.01, asterisks). (B–C) Live, isolated ankyrin-B^+/−^ cardiomyocytes display reduced (∼20%) surface expression as measured by binding of [^3^H]-ouabain (n = 3, p<0.01). (D–E) Conscious echocardiograms of wild-type and ankyrin-B^+/−^ mice before and after transverse aortic constriction. Black labels represent wild-type animals, white labels represent ankyrin-B^+/−^ data. Ankyrin-B^+/−^ mice show preserved fractional shortening compared to wild-type mice over a wide range of hemodynamic stress (induced through transverse aortic constriction).

We first compared effects of ouabain and ankyrin-B-deficiency on cellular shortening in freshly isolated unloaded wild-type and ankyrin-B^+/−^ adult ventricular cardiomyocytes. The extent of cell shortening provides a functional readout of [Ca^2+^]_i_ transients and contractility, since cardiomyocytes respond to elevated calcium levels by contraction. Field-stimulated wild-type cardiomyocytes exhibited 12.3% shortening (n = 5 mice) that increased ∼1.7-fold to 20% in the presence of 100 µM ouabain (a saturating concentration) (Figure 2A, p<0.01). In contrast, ankyrin-B^+/−^ cardiomyocytes exhibited 15% basal shortening (n = 4 mice), a value significantly greater than that of unloaded wild-type cardiomyocytes (p<0.01). Ankyrin-B^+/−^ cardiomyocytes were further activated by 100 µM ouabain to 20% shortening, or a ∼1.3-fold increase (Figure 2A, p<0.01). Untreated ankyrin-B^+/−^ cardiomyocytes thus behave as if they were treated with ouabain, with increased basal levels of contraction. In response to treatment with maximal concentrations of ouabain, wild-type and ankyrin-B^+/−^ cardiomyocytes are activated to the same final extent. These results suggested that the fraction of Na/K ATPase missing in ankyrin-B^+/−^ cardiomyocytes accounts for ∼38% of the maximal contractile response to ouabain.

There is considerable uncertainty regarding which isoforms of the Na/K ATPase are physiological targets for cardiac glycosides and in which species [28], [29]. Therefore we determined effects of ankyrin-B deficiency in mouse cardiomyocytes on the affinity and capacity for ^3^[H]-ouabain, which is a commonly used cardiac glycoside (Figure 2B–C). Freshly isolated cardiomyocytes from ankyrin-B^+/−^ mice have reduced numbers (∼15%) of surface ^3^H-ouabain-binding sites, but similar affinity for ouabain compared to cells derived from wild-type littermates (wild-type B_max _ = 1.90 pmol/mg (Kd = 31.3 nM); ankyrin-B^+/−^ B_max_ = 1.61 pmol/mg (Kd = 33.1 nM); Figure 2C). Scatchard plots reveal a single class of ouabain-binding sites for both wild-type and ankyrin-B^+/−^ cells, suggesting that the sites lost in ankyrin-B^+/−^ cells have the same affinity as the sites that remain (Figure 2C). These data with isolated cardiomyocytes are consistent with a ∼15% reduction in level of both Na/K ATPase α_1_ and α_2_ isoforms in ankyrin-B^+/−^ heart tissue determined previously [14]. Na/K ATPase expression and localization at sarcolemmal and intercalated disc membranes is unaffected in ankyrin-B^+/−^ cells [14]. Therefore, loss of T-tubule-associated Na/K ATPase is paralleled by loss of a small population of ouabain-binding sites (∼16%) in ankyrin-B^+/−^ cardiomyocytes that have the same affinity for ouabain as the residual binding sites.

### Enhanced cardiac function in ankyrin-B^+/−^ mice

The finding that loss of ankyrin-B-coupled Na/K ATPase mimics effects of cardiac glycosides in isolated ankyrin-B^+/−^ cardiomyocytes, suggested the hypothesis that ankyrin-B^+/−^ mice also have enhanced cardiac function. Digitalis has a relatively subtle effect on the unstressed heart with a normal left ventricular (LV) pressure, and at one point was believed to only act in the context of heart failure [30]. We therefore measured left ventricular (LV) fractional shortening (an intact heart correlate of cellular shortening and one measure of cardiac function) by echocardiography in conscious mice before and after induction of LV pressure overload. LV pressure overload was induced by transverse aortic constriction (TAC; [22]). Before TAC, ankyrin-B^+/−^ mice displayed a modest, but significant 10% increase in percent fractional shortening compared to wild-type mice (wild-type = 58.3±1.9%, n = 16; ankyrin-B^+/−^ = 63.9±1.5% n = 15 mice; p<0.05; Figure 2D). Following TAC for seven days that induced similar increases in LV pressure overload as measured by the trans-stenotic gradient (see Figure 2E), ankyrin-B^+/−^ mice exhibited preservation of higher fractional shortening (51.8±7.8%, n = 11; Figure 2D, p<0.05) compared to the striking ∼74% decline in fractional shortening to 29.8±7.9%, n = 9; p<0.05 observed in wild-type animals. Therefore, one benefit of loss of ankyrin-B-coupled Na/K ATPase is preservation of cardiac function under conditions of elevated afterload induced by hemodynamic stress.

### Ankyrin-B^+/−^ mice display reduced sensitivity to ouabain-induced arrhythmia

**Figure 3 pone-0001051-g003:**
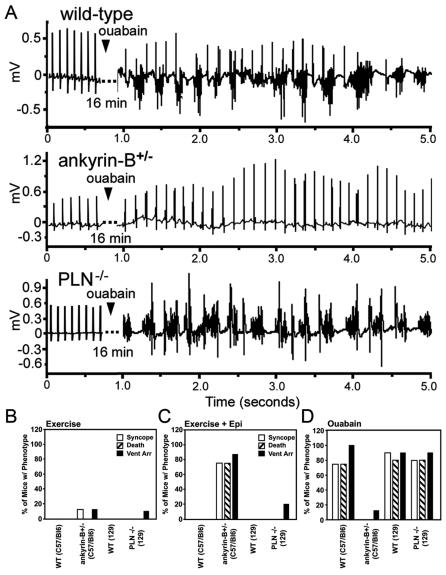
Ankyrin-B^+/−^ mice are resistant to ouabain-induced ventricular arrhythmia and death. Wild-type (n = 8) and ankyrin-B^+/−^ mice (n = 8), and wild-type (n = 10) and phospholamban-null mice (n = 10) were surgically-implanted with radiotelemetry ECG probes. Mice were injected with a high dose (10 mg/kg) of ouabain or 2 mg/kg epinephrine, and conscious ECGs were recorded. Unlike wild-type or phospholamban-null mice, ankyrin-B^+/−^ mice display resistance to ouabain-induced ventricular arrhythmia (1/8) and death (0/8). Panel A represents ECG data from wild-type, ankyrin-B^+/−^, and PLN null mice treated with ouabain. Data in panels B–D represent summary syncope, death, and arrhythmia data for mice following exercise (panel B), exercise plus epinephrine injection (panel C), and ouabain injection (panel D).

Enthusiasm for clinical use of cardiac glycosides has subsided due to their capacity to promote fatal cardiac arrhythmias. The results with isolated adult cardiomyocytes suggested the possibility that ankyrin-B^+/−^ mice might be at least partially resistant to proarrhythmic effects of ouabain. We evaluated effects of ouabain on heart rate and rhythm by measuring electrocardiograms in conscious animals using implanted wireless transmitters (Figure 3). Intraperitoneal injection of a low, sub-lethal dose of ouabain (100 µg/kg) leads to an expected modest decrease in heart rate in 6/6 wild-type mice tested, but no effect in ankyrin-B^+/−^ mice, which already have bradycardia [14]. Lethal doses of ouabain (10 mg/kg) caused polymorphic arrhythmia in wild-type C57BL/6 WT mice (Figure 3A; 8/8 mice) within 16 minutes of injection, followed by death (6/8). Identical injections of lethal doses of ouabain to C57BL/6 ankyrin-B^+/−^ littermate mice did result in decreased heart rate (8/8), but no significant ventricular arrhythmias (1/8), and, remarkably, no death (0/8; Figure 3C). Ankyrin-B^+/−^ mice thus are resistant to ouabain in three functional readouts: reduction in heart rate, ventricular arrhythmia, and death [31].

Ankyrin-B^+/−^ mice are not intrinsically resistant to all forms of arrhythmia since strenuous exercise plus injection of high doses of epinephrine caused ventricular arrhythmia, syncope, and death in ∼75% of ankyrin-B^+/−^ mice [14]. However, a trivial explanation for ouabain-resistance of ankyrin-B^+/−^ mice could be that chronic elevation of [Ca^2+^]_i_ transients in ankyrin-B^+/−^ cardiomyocytes caused compensatory adaptations. Phospholamban-null mice have chronic elevation of [Ca^2+^]_i_ transients comparable to ankyrin-B^+/−^ mice due to increased Ca-affinity of SERCA2 [32] and provide a control for generalized Ca^2+^-induced changes in protein expression. Phospholamban-null mice in a 129 background respond to high doses of ouabain with a similar arrhythmia (9/10) and death (8/10) in the same manner as C57BL/6 WT mice (8/8, 6/8) and 129 WT mice (9/10,8/10; Figure 3C). Ankyrin-B^+/−^ mice thus are resistant to ouabain-induced cardiac arrhythmia due to a mechanism independent of adaptation to chronic Ca^2+^ elevation.

### Ankyrin-B^+/−^ mice display premature senescence and reduced longevity

**Figure 4 pone-0001051-g004:**
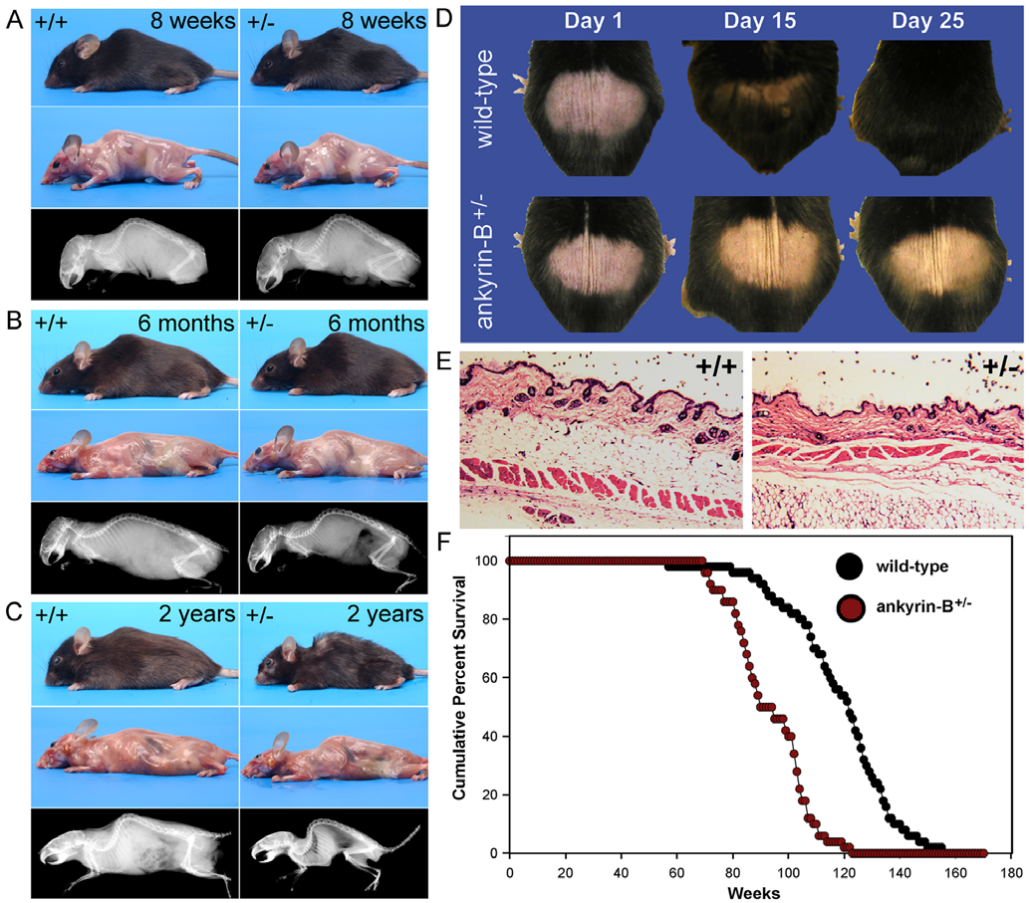
Ankyrin-B^+/−^ mice display premature senescence and reduced longevity. Gross phenotypes of wild-type and ankyrin-B^+/−^ mice at (A) eight weeks, (B) six months, and C) two years. While we observe no major differences in phenotypes at eight weeks, we observe a slight decrease in size and the presence of lordokyphosis in ankyrin-B^+/−^ mice at six months of age. At two years, unlike wild-type littermates, living ankyrin-B^+/−^ mice display a significant decrease in adiposity and loss of soft tissues as well as prominent lordokyphosis. D, Dorsal hair regrowth phenotypes in 12 month C57Bl/6 wild-type and ankyrin-B^+/−^ littermates. We observed no difference in regrowth in 1 or 3 month mice. E, H&E stain of dorsal skin sections from age-matched (24 month) wild-type and ankyrin-B^+/−^ littermates. Note the decrease in adipose from the subdermis of ankyrin-B^+/−^ mouse skin. F, Reduced longevity of ankyrin-B^+/−^ mice compared with wild-type littermates.

As ankyrin-B mutant mice aged we noticed a consistent pattern of kyphosis, hair loss and general deterioration compared to their littermates. We therefore evaluated senescence in ankyrin-B^+/−^ mice. Consistent with premature aging, we observed decreased soft tissue mass in >12 month ankyrin-B^+/−^ animals (see 12 and 24 month animals in Figure 4A–C). In fact, when compared to their age-matched wild type littermates, ankyrin-B^+/−^ mice demonstrate a loss of adiposity that begins at 6 months of age and progresses until death (Figure 4). This loss is not due to a decrease in food intake or core body temperature. These mice consume similar quantities of rodent chow, and have similar conscious body temperatures (not shown). Moreover, ankyrin-B^+/−^ mice display severe kyphosis which begins at 6 months of age and becomes increasingly pronounced in two year ankyrin-B^+/−^ mice, Figure 4A–C, *bottom panels*). Ankyrin-B^+/−^ mice (>1 yr) also have an impaired ability to re-grow hair (Figure 4D). Hair growth requires functioning stem cells, suggesting these animals may have impaired stem cell regeneration, which can promote premature senescence (Figure 4F) [33]. Histological evaluation of the dorsal skin sections of two-year old wild-type and ankyrin-B^+/−^ mice show a decrease in sub-dermal adipose tissue when compared with wild-type littermates, which also is a phenotype consistent with senescence (Figure 4E). Finally, the life-span of ankyrin-B^+/−^ mice is significantly reduced compared with wild-type littermate controls (Figure 4F). Specifically, ankyrin-B^+/−^ mice live on average to ∼90 weeks; while their wild-type littermates survive to ∼120 weeks (consistent with the C57Bl6 life-span [34]). These data demonstrate that accelerated aging is a highly penetrant consequence of ankyrin-B-deficiency in mice. A future goal will be to further explore the link between these longevity data in mice with preliminary human ankyrin-B variant data presented in Figure 1D.

### Ankyrin-B-haploinsufficiency in multiple cell types

**Figure 5:**
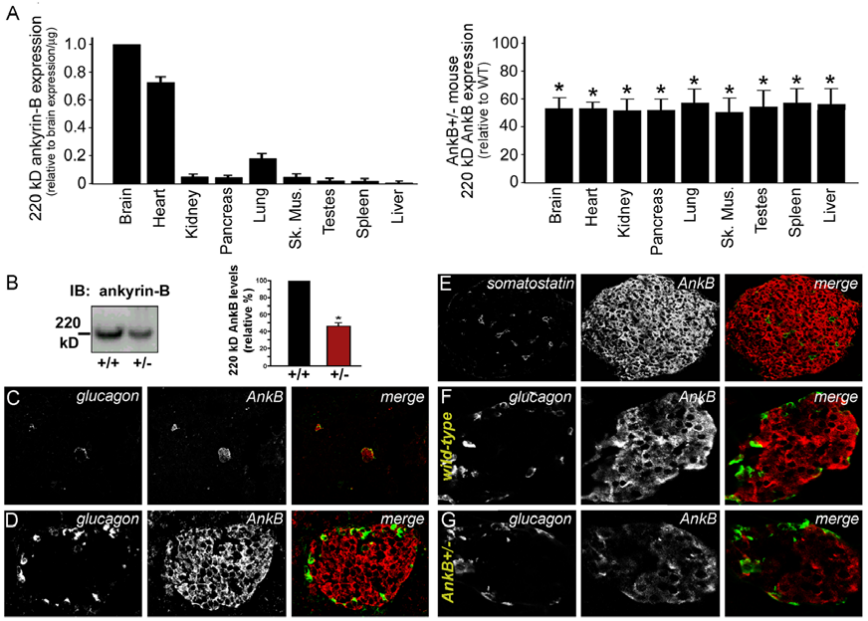
Ankyrin-B-haploinsufficiency in multiple cell types. A, Relative 220 kD ankyrin-B expression across mouse tissues. Right panel illustrates expression of 220 kD ankyrin-B in ankyrin-B^+/−^ mouse tissues compared with wild-type littermate tissue (n>3, p<0.05). B, Quantitative immunoblot of 220 kD ankyrin-B expression in wild-type and ankyrin-B^+/−^ mouse pancreas using ankyrin-B Ig. Protein levels were quantified by 125-labeled Protein A (n>3). C–D, Immunolocalization of glucagon (left) and ankyrin-B (right) in the Islet of wild-type mouse pancreas. Note that we observe no immunoreactivity of ankyrin-B in the exocrine pancreas. E, Immunolocalization of somatostatin and ankyrin-B in wild-type islets. We observe no co-expression of ankyrin-B and somatostatin in delta cells. F, Immunolocalization of glucagon and ankyrin-B in wild-type mouse islets. We observe no co-localization of glucagon and ankyrin-B in alpha cells. G, Immunolocalization of glucagon and ankyrin-B in Islets of ankyrin-B^+/−^ mice. Note the significant decrease in ankyrin-B levels.

As a first step in determining possible physiological differences underlie the complex phenotype of ankyrin-B^+/−^ mice, we evaluated in these mice the pattern of expression of ankyrin-B. 220 kD ankyrin-B is abundantly expressed in both brain, heart and thymus [12], [35]. Ankyrin-B also is present at detectable levels in skeletal muscle, kidney, lung, testes, spleen and lung, and is reduced about 50 percent in all of these tissues in ankyrin-B^+/−^ mice (Figure 5A–B).

We next determined the cell types expressing ankyrin-B in several tissues. In the pancreas, ankyrin-B is highly expressed in islets and is virtually absent from surrounding acinar cells (Figure 5C). Within pancreatic islets, ankyrin-B staining was concentrated in beta cells and virtually absent from alpha cells (glucagon-positive) or delta cells (somatostatin-positive) (Figure 5D–E). Ankyrin-B immunostaining in β-cells is significantly reduced in ankyrin-B^+/−^ pancreas (Figure 5B, 5F–G). Identical labeling and imaging protocols (see *Methods*) revealed an approximate 47% decrease in the intensity ratio of ankyrin-B/glucagon in ankyrin-B^+/−^ islets compared to wild-type islets (+/+, 0.90±0.1; +/−, 0.48±0.1, n = 20, p<0.05). As ankyrin-B expression in acinar cells is minimal, the reduction of ankyrin-B by quantitative immunoblot in pancreas (Figure 5B) is due primarily to loss of ankyrin-B in beta cells.

In the heart, reduced expression of ankyrin B protein results in decreased levels of ankyrin-binding partners IP3 receptor, Na/Ca exchanger and Na/K ATPase. The loss of these channels/transporters in critical microdomains of cardiomyocyte transverse-tubules/sarcoplasmic reticulum is believed to be the basis of the arrhythmia phenotype. [16]. We determined that ankyrin-B haploinsufficiency also results in loss of IP3 receptor in other cell types, including Purkinje neurons in the cerebellum and bronchial epithelial cells in the lung as a correlate of ankyrin-B function in these cells (Figures S1,S2,S3). Future work will investigate the consequences of ankyrin-B deficiency at a molecular level, and determine which cell types contribute to the global phenotype of the ankyrin-B mouse.

## Discussion

This study reports that certain loss-of-function variants in the *ANK2* gene are present in “normal” European and west African populations. The frequency of these variants is surprising given the high degree of protein conservation between species and within most human populations. Mice heterozygous for a null mutation in ankyrin-B have previously been demonstrated to have a similar cardiac arrhythmia as humans with ankyrin-B variants [14], [15]. Further characterization of ankyrin-B^+/−^ mice in this paper reveal a heretofore unrecognized syndrome of increased cardiac contractility and resistance to cardiac glycoside-induced arrhythmia that is balanced by premature senescence. Ankyrin-B is co-expressed with IP3 receptors in specific cell types including Purkinje neurons and bronchial epithelial cells. Interestingly, both ankyrin-B and IP3 receptors are reduced in these cells in ankyrin-B^+/−^ mice (Figures S1,S2,S3). Diminished function of IP3 receptors in calcium signaling thus may underlie some of the phenotypes observed in ankyrin-B^+/−^ mice. Together, these findings suggest that the 2 percent of Europeans and up to 8 percent of individuals in west Africa that harbor specific ankyrin-B variants may respond favorably to digitalis treatments and also have increased risk of sudden cardiac death and diseases related to aging.

Results with ankyrin-B^+/−^ mice suggest several hypotheses that will be important to test in people. The high levels of ankyrin-B expression in the brain and pancreatic beta cells and early senescence in ankyrin-B^+/−^ mice suggests that human populations with age-related neurological disorders and/or type 2 diabetes should be evaluated for *ANK2* variants. Resistance of ankyrin-B^+/−^ mice to ouabain-induced arrhythmia suggests that cardiac glycosides could be potentially used more safely in humans with ankyrin-B variants. Moreover, these findings suggest that interference with ankyrin-B function in the heart could be a potentially useful strategy to modify cardiac contractility.

8.6 percent of the people in our sample from Ghana are heterozygous for the L1622I gene variant. Therefore up to 0.7 percent of this population could be homozygous for the L1622I mutation in the absence of negative selection. While no individuals homozygous for the L1621I variant were detected in 384 individuals, this could result from the relatively small sample size. It will be of interest in the future to screen larger populations from Ghana as well as African Americans to determine if homozygous L1622I individuals exist, and if so whether they exhibit features of ankyrin-B syndrome characterized so far in mice.

An obvious question is the nature of the benefit conveyed by certain ankyrin-B variants. Resistance to malaria is an unlikely factor, since ankyrin-B is not expressed in erythrocytes (not shown), or in vascular endothelial cells (Figure S2), and since variants are present in both Africans and Europeans. A simple hypothesis is that increased cardiac contractility enhances cardiac output during physical exertion, and thus improves survival. A corollary that can be experimentally tested is that athletes may be more likely to have ankyrin-B variants. Moreover, ankyrin-B variants may contribute to sudden cardiac death among young athletes.

A striking and highly penetrant effect of ankyrin-B deficiency in the mouse is a moderate reduction in life span accompanied by premature senescence in multiple tissues. One hypothesis to explain this phenotype is that disordered calcium homeostasis as observed in ankyrin-B^+/−^ cardiomyocytes [14] results in mitochondrial stress. Inter-relationships between calcium, mitochondria, and aging have been attributed to accumulated damage from reactive oxygen due to calcium overload of mitochondria [36]. It will be important to evaluate effects of ankyrin-B deficiency and loss-of-function variants on mitochondrial function.

While the ankyrin-B^+/−^ mouse model has been instrumental in demonstrating the role of ankyrin-B for normal vertebrate physiology (Figures 2–5; [14]–[16], [35]), the broad tissue distribution of ankyrin-B (see Figure 5) has greatly complicated the ability to precisely correlate animal phenotypes with specific tissue/cell dysfunction. For example, defects in dorsal hair regrowth in ankyrin-B^+/−^ mice (Figure 4D) are indicative of impaired stem cell regeneration. However, since ankyrin-B is also expressed in thymus [35], an alternative hypothesis is that reduced ankyrin function in thymus results in reduced resistance to infection or even potential auto-immune defects associated with hair loss. A central future focus for the field will be to use genetically-engineered mouse and cell models to identify which specific cell types are responsible for the global phenotypes observed in the ankyrin-B^+/−^ mouse.

In summary, this study demonstrates an unexpected prevalence of mutations in ankyrin-B that have been linked to risk for sudden cardiac death. We present the first evidence from a mouse model deficient in ankyrin-B that ankyrin-B variants can be viewed as balanced variants with increased risk of sudden death and early aging offset by increased cardiac contractility.

## Supporting Information

Figure S1 InsP3R expression in Purkinje neurons is reduced in ankyrin-B+/− brain. Cerebellar sections from wild-type and ankyrin-B+/− mice were immunolabeled with affinity-purified antibody to (A) ankyrin-B and (B) InsP3R (pan). Wild-type and ankyrin-B +/− sections were prepared, stained, and imaged using identical protocols. Scale bar equals 50 microns.
(5.10 MB TIF)

Figure S2 Ankyrin-B expression at the apical membrane of airway epithelial cells is significantly reduced in ankyrin-B+/− lung. Lung sections from wild-type and ankyrin-B+/− mice were immunolabeled with affinity-purified antibodies for beta-tubulin and ankyrin-B. No staining was observed using non-immune serum. Scale bars from top to bottom equal 100, 30, 100, and 25 microns. Wild-type and ankyrin-B+/− tissue sections were prepared and imaged identically. Abbreviations: Bronchus (Br), apical (ap), smooth muscle (sm), alveoli (al), artery (A).
(3.19 MB TIF)

Figure S3 InsP3R expression is exclusively reduced in ankyrin-B+/− lung where co-expressed with ankyrin-B. InsP3R localization with co-labeling of beta-tubulin (columnar epithelial cells) in wild-type and ankyrin-B+/− lung sections. InsP3R (pan InsP3R) is highly expressed at the apical membrane of epithelial cells and in smooth muscle. No staining was observed using pre-immune serum. Bottom, reduction of ankyrin-B in ankyrin-B +/− lung is associated with InsP3R reduction at the apical membrane of epithelial cells, while there is no reduction in smooth muscle InsP3R. Scale bars from top to bottom equal 100, 20, 100, and 15 microns. Wild-type and ankyrin-B +/− tissue sections were prepared and imaged identically. Abbreviations: Bronchus (Br), apical (ap), smooth muscle (sm), alveoli (al), artery (A).
(3.17 MB TIF)
