# Supplementary figures and images for: Ankyrin-B Syndrome: Enhanced Cardiac Function Balanced by Risk of Cardiac Death and Premature Senescence
Source: PLoS One. 2007 Oct 17;2(10):e1051. doi: 10.1371/journal.pone.0001051 (PMC2013943; doi:10.1371/journal.pone.0001051)

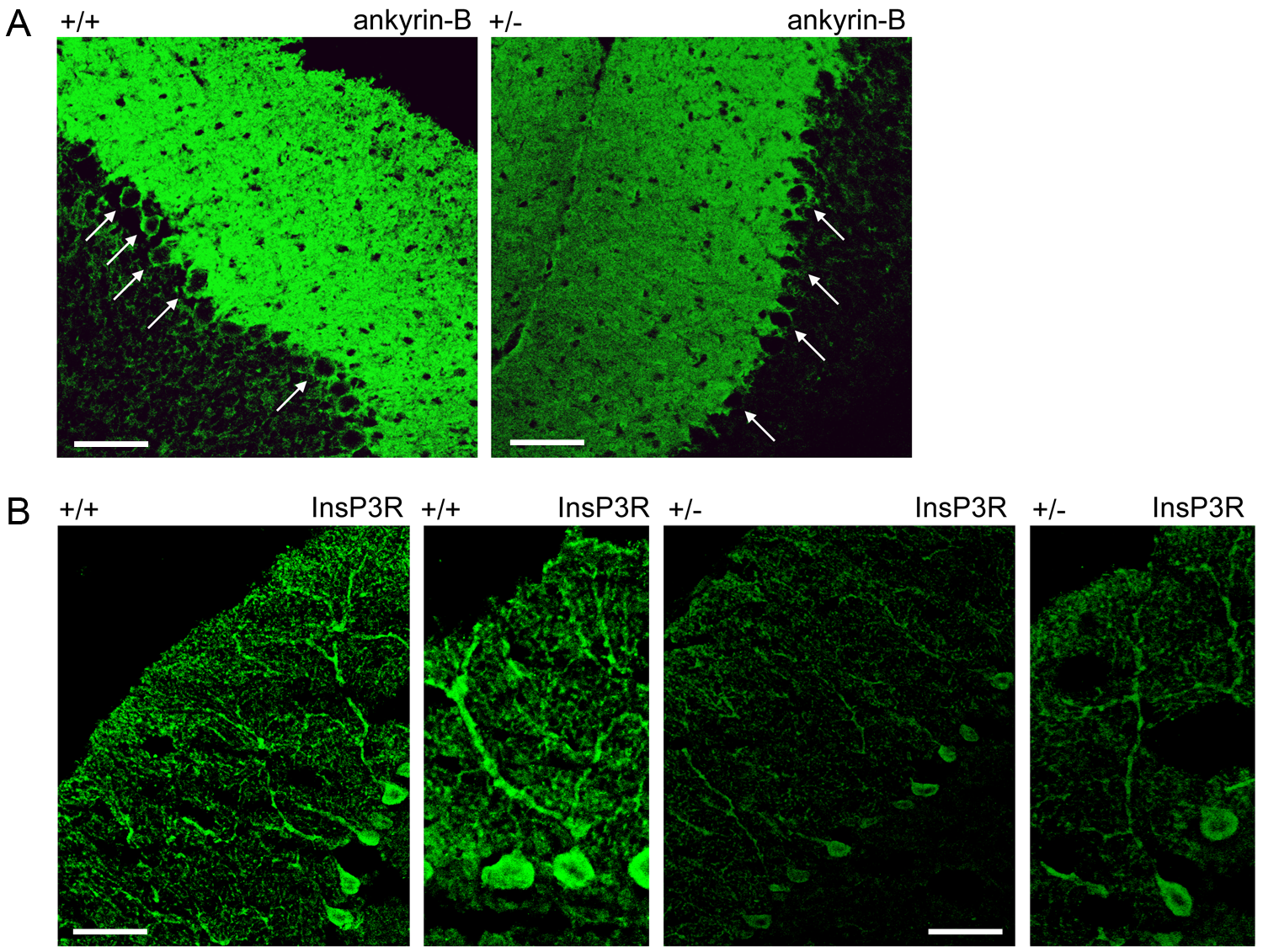

Supplement: Figure S1 — InsP3R expression in Purkinje neurons is reduced in ankyrin-B+/− brain. Cerebellar sections from wild-type and ankyrin-B+/− mice were immunolabeled with affinity-purified antibody to (A) ankyrin-B and (B) InsP3R (pan). Wild-type and ankyrin-B +/− sections were prepared, stained, and imaged using identical protocols. Scale bar equals 50 microns. (5.10 MB TIF) [file pone.0001051.s001.tif]

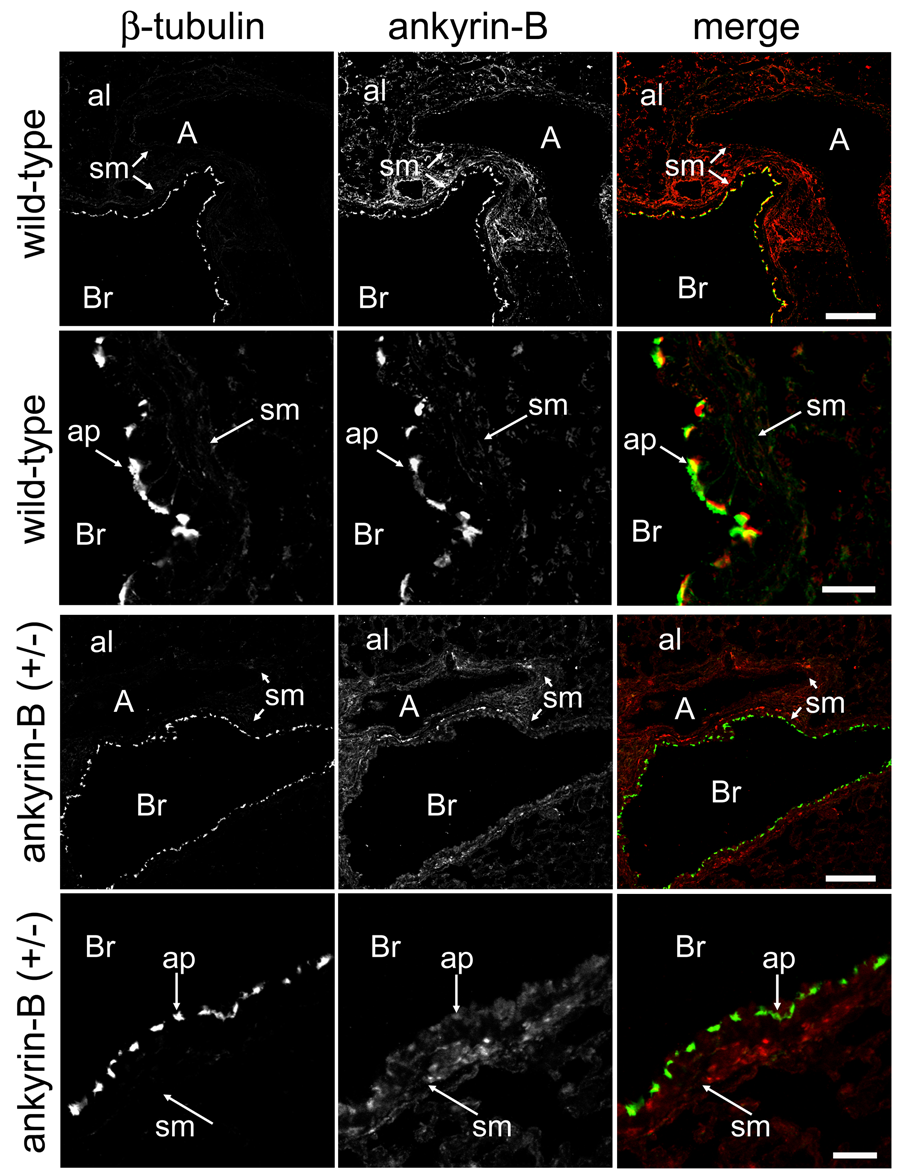

Supplement: Figure S2 — Ankyrin-B expression at the apical membrane of airway epithelial cells is significantly reduced in ankyrin-B+/− lung. Lung sections from wild-type and ankyrin-B+/− mice were immunolabeled with affinity-purified antibodies for beta-tubulin and ankyrin-B. No staining was observed using non-immune serum. Scale bars from top to bottom equal 100, 30, 100, and 25 microns. Wild-type and ankyrin-B+/− tissue sections were prepared and imaged identically. Abbreviations: Bronchus (Br), apical (ap), smooth muscle (sm), alveoli (al), artery (A). (3.19 MB TIF) [file pone.0001051.s002.tif]

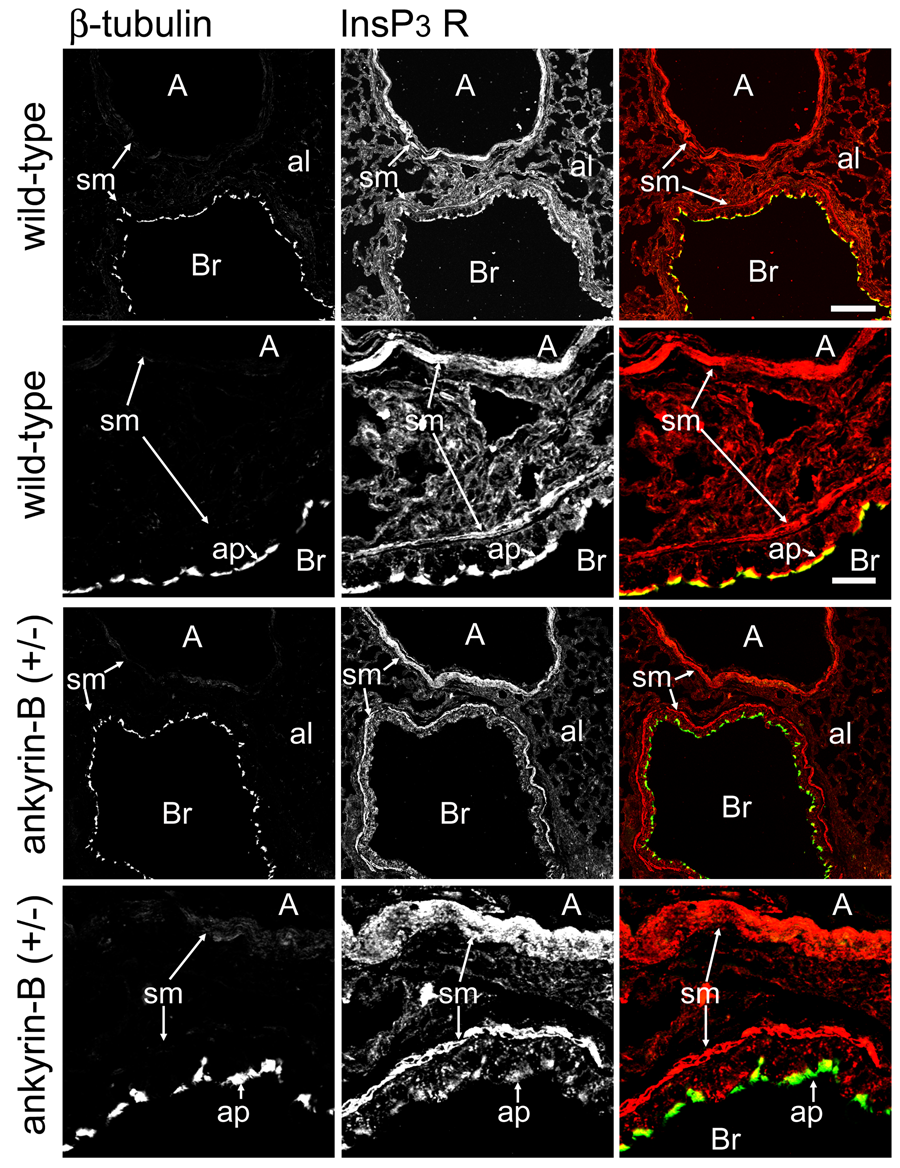

Supplement: Figure S3 — InsP3R expression is exclusively reduced in ankyrin-B+/− lung where co-expressed with ankyrin-B. InsP3R localization with co-labeling of beta-tubulin (columnar epithelial cells) in wild-type and ankyrin-B+/− lung sections. InsP3R (pan InsP3R) is highly expressed at the apical membrane of epithelial cells and in smooth muscle. No staining was observed using pre-immune serum. Bottom, reduction of ankyrin-B in ankyrin-B +/− lung is associated with InsP3R reduction at the apical membrane of epithelial cells, while there is no reduction in smooth muscle InsP3R. Scale bars from top to bottom equal 100, 20, 100, and 15 microns. Wild-type and ankyrin-B +/− tissue sections were prepared and imaged identically. Abbreviations: Bronchus (Br), apical (ap), smooth muscle (sm), alveoli (al), artery (A). (3.17 MB TIF) [file pone.0001051.s003.tif]
